# Measures of mortality among people living with fibrodysplasia ossificans progressiva in the United States, 2017-2023

**DOI:** 10.1093/jbmrpl/ziag154

**Published:** 2026-09-15

**Authors:** Alexander Breskin, Victor O Popoola, Michelle Davis, Sammi Kile, Amal Souttou, Susan Rhee, Caitlin Knox

**Affiliations:** Pharmacoepidemiology, Regeneron Pharmaceuticals, Inc., Tarrytown, NY 10591, United States; Pharmacoepidemiology, Regeneron Pharmaceuticals, Inc., Tarrytown, NY 10591, United States; International Fibrodysplasia Ossificans Progressiva Association, North Kansas City, MO 64116, United States; International Fibrodysplasia Ossificans Progressiva Association, North Kansas City, MO 64116, United States; Pharmacoepidemiology, Regeneron Pharmaceuticals, Inc., Tarrytown, NY 10591, United States; Pharmacoepidemiology, Regeneron Pharmaceuticals, Inc., Tarrytown, NY 10591, United States; Pharmacoepidemiology, Regeneron Pharmaceuticals, Inc., Tarrytown, NY 10591, United States

**Keywords:** mortality rate, survival analysis, observational study, fibrodysplasia ossificans progressiva, rare disease

## Abstract

Fibrodysplasia ossificans progressiva (FOP) is an ultra-rare genetic disease characterized by heterotopic ossification in connective tissues. Few epidemiologic studies have investigated mortality rates among people with FOP. We conducted a retrospective cohort study to understand mortality in people with FOP. Data were obtained from people with FOP in the United States (US) who participated in either the International FOP Association (IFOPA) membership database or the IFOPA patient registry between January 1, 2017, and December 31, 2023. From 2017 to 2023, 250 people with FOP were identified. The age-adjusted mortality rate was 34.6 (95% CI: 22.5-51.7) deaths per 1000 person-years. The annual age-adjusted mortality rate ranged from 21.0 (95% CI: 3.2-77.8) to 51.6 (95% CI: 19.1-114.7) deaths per 1000 person-years; the annual age-adjusted mortality rate ranged from 7.2 to 8.9 per 1000 person-years in the general US population. The median age at death for people with FOP was 50.5 (95% CI: 46.5-57.5) yr vs 80.5 yr in the general US population. The restricted mean time lost for people with FOP was 32.3 (95% CI: 26.8-37.8) yr vs 8.1 yr in the general US population. The US FOP population has substantial excess mortality vs the general US population.

## Graphical Abstract

**Graphical Abstract:**
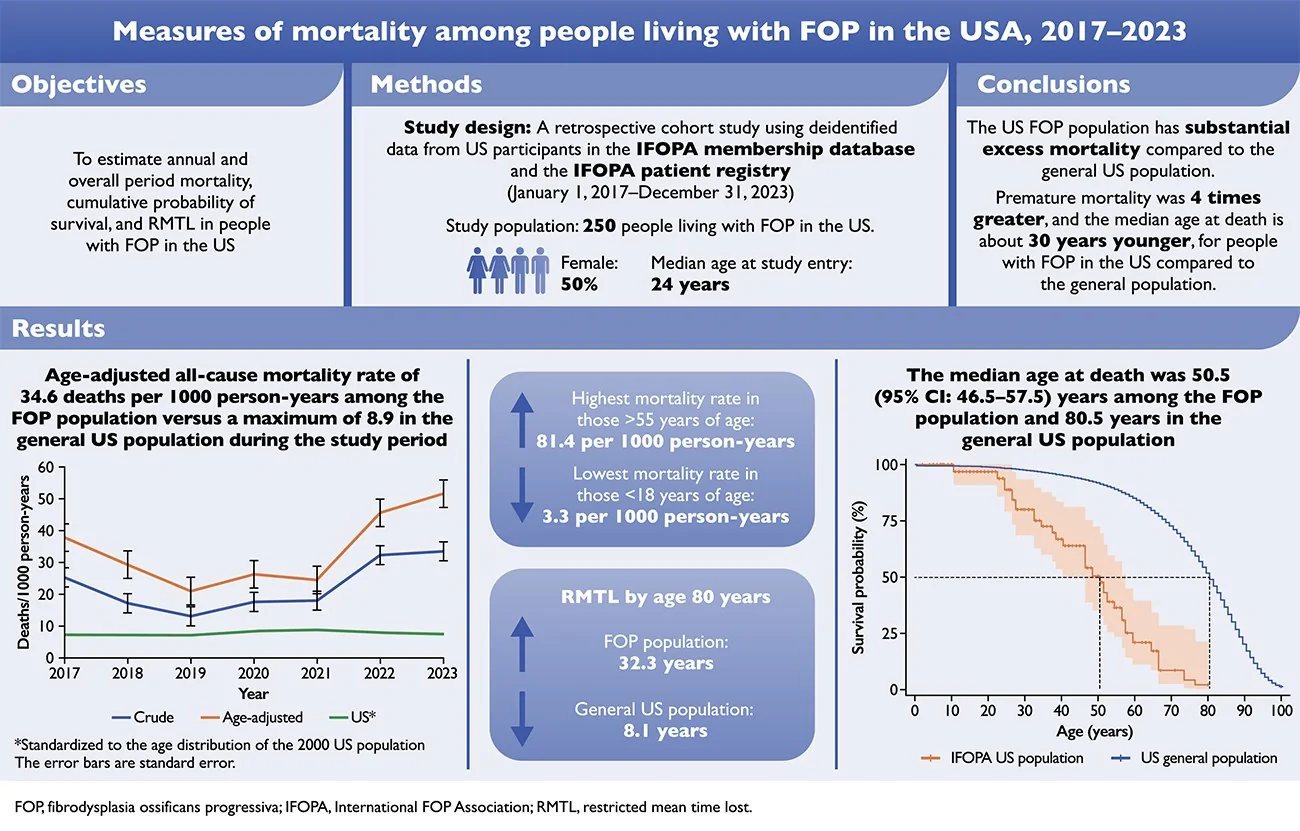

## Introduction

Fibrodysplasia ossificans progressiva (FOP) is an ultra-rare genetic disease, with a reported adjusted prevalence estimate of 0.88 per million people in the United States (US).^1^ Fibrodysplasia ossificans progressiva is a progressively debilitating disease that markedly impacts the lifespan and quality of life of affected individuals.^1^^,^^2^ Fibrodysplasia ossificans progressiva first presents with congenital bilateral deformities in the big toe and is characterized by the development of heterotopic ossification of soft tissues.^2^^,^^3^ Heterotopic ossification may occur spontaneously or following trauma, and may be preceded by episodic, painful, and soft-tissue swelling known as flare-ups.^4^ Diagnosis of FOP is often based on clinical features; however, molecular genetic testing demonstrating mutations of the activin A receptor type I (*ACVR1*) gene is confirmatory for a FOP diagnosis.^5^

In August 2023, the U.S. Food and Drug Administration (FDA) approved palovarotene for reducing the volume of new heterotopic ossification associated with FOP for adults, female children aged ≥8 yr, and male children aged ≥10 yr.^6^ In August 2026, garetosmab, a human monoclonal antibody that blocks activin A, was approved to reduce formation of new heterotopic ossification lesions and clinician-assessed flare-ups in adults with FOP in the US.^7^ Management options for FOP are limited and historically focused on the management of flare-ups. Despite positive treatment developments, no curative intervention currently exists for FOP.^5^^,^^8^

Information on measures of mortality among people living with FOP is limited. Literature commonly references anecdotal evidence of an excess mortality risk, but only 2 studies in the last 2 decades have characterized mortality in the FOP population.^2^^,^^9^

In 2010, Kaplan et al.^2^ reported 60 deaths among people living with FOP from 1973 to 2006. The median age at death was 40 yr, and the calculated median lifespan was 56 yr (95% CI: 51-60) from 371 members of the International FOP Association (IFOPA) who were alive in January 2006 as well as from the 60 deaths identified during the 33-yr study period. The deaths were obtained from the IFOPA and the International FOP Clinic at the University of Pennsylvania. Of the 48 deaths where cause was determined, 54% were from cardiorespiratory failure secondary to thoracic insufficiency syndrome, and 15% were due to pneumonia. Importantly, 30% of people living with FOP survived to the age of 60 yr, compared with >80% of people in the US population in 1980. Mortality rates were not reported.

She et al.^9^ analyzed the mortality rate and risk factors for mortality in a retrospective cohort of 65 people living with FOP in China. Over the 10-yr study period, the overall mortality rate was 13.8%; it was 11.1% for those aged ≤18 yr at diagnosis; and 20.0% for those aged >18 yr. Serum osteocalcin level was negatively correlated with mortality in those aged ≤18 yr at the time of their FOP diagnosis.

Both estimates, however, have limitations that constrain their applicability to the contemporary US FOP population. Kaplan et al.^2^ combined deaths ascertained from 2 sources with a denominator drawn from a single cross-section of members living in 2006, without accounting for delayed entry into observation, while She et al.^9^ reported a crude cumulative proportion of deaths in a Chinese cohort over variable follow-up. To our knowledge, no prior study in the US FOP population has estimated mortality in the same underlying population as the deaths while accounting for left truncation. We therefore conducted a retrospective cohort study to estimate annual and period mortality, cumulative survival, and restricted mean time lost (RMTL) among people living with FOP in the US.

## Materials and methods

### Study design, data sources, and study population

We used deidentified data from the US IFOPA membership database and US participants of the IFOPA patient registry from January 1, 2017, until December 31, 2023, to characterize mortality in the US FOP population. The IFOPA membership database collects diagnosis information (eg, physician confirmation of diagnosis and date of diagnosis) along with other demographic information at the individual level. The IFOPA also has a patient registry that tracks the treatment and health information of people living with FOP across the globe; it is the largest FOP patient registry in the US. At the time of enrollment in the patient registry, individuals provide informed consent to the IFOPA for research activities. The IFOPA has regular contact with most known people living with FOP in the US, and confirms the vital status of its members. To facilitate the current study, the IFOPA contacted members to confirm their vital status over an 8-mo period. The data for the current study were extracted by the IFOPA and provided as a deidentified, aggregated dataset. This study of deidentified data was deemed exempt from review by an institutional review board.

### Study population

Our study included all people living with FOP in the US in either data source from January 1, 2017, until December 31, 2023, deduplicated to ensure each person appeared in the data only once. People whose primary residence was not in the US were excluded from the analysis. All study variables were derived from the existing data obtained from the data sources as collected in prespecified patient survey collection forms.

Study follow-up began on January 1, 2017, or June 30 of a person’s year of birth, whichever occurred first. Follow-up ended on December 31, 2023, or June 30 of the person’s year of death or loss to follow-up, whichever occurred first.

### Outcome ascertainment, covariate definitions, and statistical methods

Fibrodysplasia ossificans progressiva–related deaths were identified based on the evidence of death in either data source. Annual mortality was calculated as the number of deaths in a calendar year divided by the person-time contributed by people living with FOP during that year. Mortality cumulative incidence was estimated using the Kaplan–Meier method. The annual FOP mortality rate and risk estimates were additionally age-adjusted to the age distribution of the US census population in 2000.

Using age as the timescale, the survival curve was estimated using an extended Kaplan–Meier estimator to account for left truncation due to late entry and right-censoring due to loss of follow-up. RMTL, a measure of the average number of years of life lost by individuals up to a specific age,^10^ was calculated as the area above the Kaplan–Meier survival curve through age 80 yr (chosen based on the average life expectancy in the US). Median age at death was estimated from the survival curve.

Age was assessed as of January 1 of each calendar year, categorized as 0-18, 19-34, 35-44, 45-54, and ≥55 yr. Sex was operationalized as male, female, or not reported.

For comparison, annual mortality, RMTL, and median age at death were computed in the general US population using reported mortality rates from the Centers for Disease Control and Prevention (CDC), along with 2021 US life tables.^11^^,^^12^ Annual mortality estimates for the US general population^12^^,^^13^ and the population of people living with FOP were age-standardized using direct standardization to the US standard population in 2000,^14^ the same population used for age standardization for mortality reporting by the CDC.^12^

## Results

A total of 250 people living with FOP were identified in the IFOPA membership database and registry during the study period. The median age was 24 yr (range: 0-76; IQR: 11-37) at cohort entry, and 50% of participants were female. Eleven births and 35 deaths were recorded.

The crude mortality rate was 21.9 deaths per 1000 person-years (95% CI: 15.3-30.5), and the age-adjusted rate was 34.6 (95% CI: 22.5-51.7) (Table 1). The corresponding crude and age-adjusted cumulative incidences were 14.4% (95% CI: 9.8%-18.6%) and 20.3% (95% CI: 13.5%-26.6%), respectively. Annual crude mortality rates ranged from 13.1 (95% CI: 2.7-38.3) in 2019 to 33.5 (95% CI: 13.5-69.0) in 2023. Annual age-adjusted mortality was highest in 2023 (51.6 deaths per 1000 person-years [95% CI: 19.1-114.7]) and lowest in 2019 (21.0 [95% CI: 3.2-77.8]). By contrast, the age-adjusted annual mortality rates in the general US population ranged from 7.2 deaths per 1000 person-years in 2018 and 2019 to 8.9 in 2021.^12^^,^^13^

**Table 1:** Annual mortality among people in the US living with FOP and the general US population (2017-2023).

**Year**	**Rate, deaths/1000 person-years (95% CI)**			**Cumulative incidence, % (95% CI)**		
	**Crude**	**Age-adjusted** ^**a**^	**US** ^12^ ^,^ ^13^	**Crude**	**Age-adjusted** ^**a**^	**US** ^12^ ^,^ ^13^
**Overall**	21.9 (15.3-30.5)	34.6 (22.5-51.7)	–	14.4 (9.8-18.6)	20.3 (13.5-26.6)	–
**2017**	25.3 (9.3-55.1)	37.8 (11.7-96.0)	7.3	2.5 (0.9-5.4)	3.7 (1.2-9.2)	0.7
**2018**	17.2 (4.7-44.0)	29.3 (5.8-91.2)	7.2	1.7 (0.5-4.3)	2.9 (0.6-8.7)	0.7
**2019**	13.1 (2.7-38.3)	21.0 (3.2-77.8)	7.2	1.3 (0.3-3.8)	2.1 (0.3-7.5)	0.7
**2020**	17.6 (4.8-45.1)	26.3 (5.4-87.0)	8.4	1.7 (0.5-4.4)	2.6 (0.5-8.3)	0.8
**2021**	18.0 (4.9-46.0)	24.5 (5.7-73.9)	8.9	1.8 (0.5-4.5)	2.4 (0.6-7.1)	0.9
**2022**	32.3 (13.0-66.6)	45.6 (16.6-104.7)	8.0	3.2 (1.3-6.4)	4.5 (1.6-9.9)	0.8
**2023**	33.5 (13.5-69.0)	51.6 (19.1-114.7)	7.5	3.3 (1.3-6.7)	5.0 (1.9-10.8)	0.7

a Standardized to the age distribution of the US population in 2000.

Abbreviations: FOP, fibrodysplasia ossificans progressiva; US, United States.

Mortality among the FOP population across the entire study period varied by age (Table 2). Among age groups with any observed deaths, mortality was highest among people aged ≥55 yr (81.4 deaths per 1000 person-years [95% CI: 37.2-154.6]; cumulative incidence: 42.9% [95% CI: 17.2%-60.5%]), and lowest among those aged ≤18 yr (3.3 deaths per 1000 person-years [95% CI: 0.4-11.8]; and cumulative incidence: 2.3% [95% CI: 0.0%-5.3%]).

**Table 2:** Mortality among people in the US living with FOP by age group (2017-2023).

**Age group, yr**	**Individuals, *n***	**Person-years**	**Deaths, *n***	**Mortality rate per 1000 person-years (95% CI)**	**Cumulative incidence, % (95% CI)**
**Any**	250	1596.0	35	21.9 (15.3-30.5)	14.4 (9.8-18.6)
**0-18**	93	612.0	2	3.3 (0.4-11.8)	2.3 (0.0-5.3)
**19-34**	85	552.5	11	19.9 (9.9-35.6)	13.0 (5.5-19.9)
**35-44**	26	173.0	4	23.1 (6.3-59.2)	15.4 (0.3-28.2)
**45-54**	25	148.0	9	60.8 (27.8-115.4)	37.3 (14.6-53.9)
**≥55**	21	110.5	9	81.4 (37.2-154.6)	42.9 (17.2-60.5)

Abbreviations: FOP, fibrodysplasia ossificans progressiva; US, United States.

The median age at death was 50.5 yr (95% CI: 46.5-57.5) among the FOP population and 80.5 yr in the general US population (Figure 1).^11^ The RMTL by age 80 yr was 32.3 (95% CI: 26.8-37.8) yr for the FOP population and 8.1 yr for the general US population.^11^

**Figure 1:**
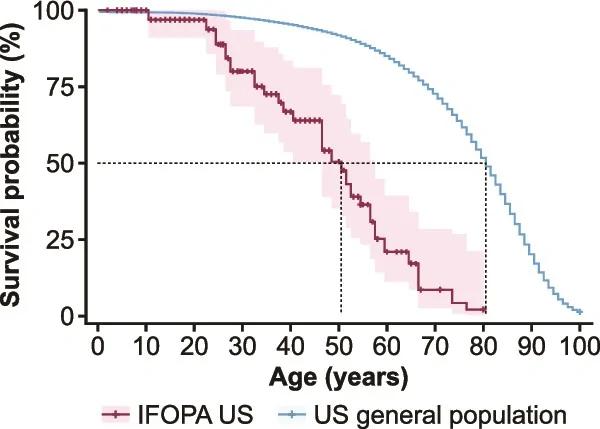
Survival among people in the US living with FOP and the general US population (2017-2023)**.** US survival curve based on 2021 Centers for Disease Control life table data.^11^ FOP, fibrodysplasia ossificans progressiva; IFOPA, International FOP Association; US, United States.

## Discussion

In this observational study, we found substantial excess mortality in the US FOP population compared with the US general population, regardless of the measure of mortality used. Among people living with FOP, annual age-adjusted mortality rates were up to 5-fold higher, median age at death was 30 yr younger, and RMTL by age 80 yr was 4 times higher than in the general US population. These descriptive findings suggest that FOP is associated with a marked increase in mortality.

Age-adjusted annual mortality in our study ranged from 21.0 to 51.6 deaths per 1000 person-years, and median age at death was 50.5 yr. The mortality rates during the study period ranged from 3.3 in people aged <18 yr to 81.4 in those aged ≥55 yr. Although current literature on mortality among people living with FOP is limited, previous reports have also suggested higher-than-average mortality in the FOP population. In the study by She et al.^9^ mortality was estimated to be 13.8% over a 10-yr period, and average age at death was 22.8 yr.^9^ However, the reported mortality is a crude proportion of deaths over a variable and largely short follow-up period, rather than a lifespan- or rate-based estimate; it was also derived in a Chinese cohort, and differences in healthcare systems and clinical practice may further limit its comparability to the US FOP population. Excess and premature mortality were also reported by Kaplan et al.^2^ in the larger cohort study. We observed a lower median age at death compared with the study by Kaplan et al. (50.5 yr [95% CI: 46.5-57.5] vs 56 yr [95% CI: 51-60]); however, the CIs from the 2 studies do overlap. Possible explanations of the differences may be due to biases such as insufficient handling of left truncation, misalignment of the populations contributing to the deaths and those contributing to person-time, and the exclusion of patients lost to follow-up prior to the conduct of the study, though the net impact of these biases is unclear.

We also found high premature mortality in our study population. Our RMTL estimate of 32.3 yr means that, over a duration of 80 yr, the average person living with FOP is expected to lose more than 30 yr of life, compared with a loss of 8.1 yr in the general population. This suggests most of the excess mortality in people living with FOP occurs relatively early in the life course. Kaplan et al.^2^ estimated that excess mortality in people living with FOP, relative to the general US population, begins around the age of 30 yr. In the study by She et al.^9^ 50% of the cohort died before the age of 23 yr. Our finding of a median survival of 50.5 yr in this cohort, compared with 80.5 yr in the general US population, along with our multi-pronged approach at quantifying premature mortality, further underlines the burden of premature mortality in the FOP population. Our results demonstrate the importance of estimating premature mortality, especially among people living with rare diseases for whom mortality rates might be harder to interpret due to small numbers.

Our study has several limitations. First, the rarity of FOP puts an inevitable restriction on sample size, leading to estimates that are imprecise and that may not fully encompass the age distribution of the FOP population. However, based on prior published research on the US prevalence of FOP,^1^ we believe we captured the majority of people in the US diagnosed with FOP within the study period. Second, the data only included the year of birth and death for each person, precluding an exact accounting of person-time. Additionally, although applied consistently, our assumption that all events occurred mid-year may inaccurately estimate person-time. Third, not all individuals were enrolled in the IFOPA membership database or the IFOPA patient registry at birth, which introduces left truncation, meaning individuals who died before having the opportunity to enroll in the study are not represented. Though our analytic approach addressed left truncation, it assumes truncation is non-informative (ie, age at enrollment is not associated with survival beyond that age). If this assumption does not hold, residual bias may remain. Finally, cause of death was not available, nor were data on comorbidities affecting the study population, thus preventing further investigation into both potential secondary conditions leading to excess mortality in this population, and quality- or health-adjusted estimates of life expectancy and survival.

## Conclusions

We found substantial excess mortality among those living with FOP as compared with the US general population. Our findings provide contemporaneous estimates on mortality for people with this rare disease, as well as providing objective evidence of excess mortality within this rare disease population.

## Data Availability

Qualified researchers may request access to study documents (including the study protocol with any amendments) that support the methods in this manuscript. Submit requests to https://vivli.org/.
